# Supplementary material for: Biological Invasions Affect Resource Processing in Aquatic Ecosystems: The Invasive Amphipod Dikerogammarus villosus Impacts Detritus Processing through High Abundance Rather than Differential Response to Temperature
Source: Biology (Basel). 2023 Jun 7;12(6):830. doi: 10.3390/biology12060830 (PMC10295368; doi:10.3390/biology12060830)
Supplement: Supplementary file 1 [file biology-12-00830-s001.zip › biology-2401795-supplementary.pdf]

Supplementary table S1. Parametric coefficients of GAM for shredding experiment

Factor	Estimate	SE	t value	p value
D. villosus vs *G. pulex*	-1.20	0.10	-12.09	<0.001
Unparasitised *G. pulex* vs *D. villosus*	-0.27	0.10	-2.70	0.007
Parasitised *G. pulex* vs *D. villosus*	-0.40	0.10	-3.90	<0.001
Temperature	-0.05	0.01	-8.81	<0.001
Unparasitised *G. pulex* x temp	-0.02	0.01	-2.33	0.02
Parasitised *G. pulex* x temp	0.01	0.01	0.59	0.56
